# Quantitative phase microscopy for non-invasive live cell population monitoring

**DOI:** 10.1038/s41598-021-83537-x

**Published:** 2021-02-24

**Authors:** Sherazade Aknoun, Manuel Yonnet, Zied Djabari, Fanny Graslin, Mark Taylor, Thierry Pourcher, Benoit Wattellier, Philippe Pognonec

**Affiliations:** 1 Phasics, Bâtiment Explorer, Espace Technologique, Route de l’Orme des Merisiers, 91190 St Aubin, France; 2 Transporter in Imaging and Radiotherapy in Oncology (TIRO), Institut des Sciences et Biotechnologies du Vivant Frédéric Joliot, CEA, School of Medicine, 28 Av de Valombrose, 06107 Nice, France; 3 HH Wills Physics Laboratory, University of Bristol, Tyndall Avenue, Bristol, BS8 1TL UK

**Keywords:** High-throughput screening, Imaging, Microscopy, Biological techniques

## Abstract

We present here a label-free development based on preexisting Quantitative Phase Imaging (QPI) that allows non-invasive live monitoring of both individual cells and cell populations. Growth, death, effect of toxic compounds are quantified under visible light with a standard inverted microscope. We show that considering the global biomass of a cell population is a more robust and accurate method to assess its growth parameters in comparison to compiling individually segmented cells. This is especially true for confluent conditions. This method expands the use of light microscopy in answering biological questions concerning live cell populations even at high density. In contrast to labeling or lysis of cells this method does not alter the cells and could be useful in high-throughput screening and toxicity studies.

## Introduction

The in vitro growth rate of cells is a good reflection of the cells’ physiological status. Primary cells in culture grow for a limited number of divisions and ultimately enter a quiescent state ("senescence") when they flatten and stop growing. On the other hand, immortalized cell lines, which represent the vast majority of cells used by biologists, can divide “forever” under the right conditions, making them valuable tools to investigate molecular mechanisms and cell physiology. These cells respond to changes in the conditions of culture with changes in their growth and/or phenotype. This is why cell culture is very useful to study differentiation, response to drugs or any kind of treatment. The molecular details of these potential modifications are investigated with molecular biology approaches performed on lysates prepared from these cells. Monitoring cell growth and phenotype by time-lapse microscopy could be a powerful way to find out whether a particular treatment results in some detectable cell growth or phenotypic differences throughout time. This requires both qualitative and quantitative imaging strategies. Classical microscopy of live cells under visible light does not allow convenient quantification. Expression of fluorescent markers such as fusion proteins with fluorescent tags, or fluorescent chemicals such as the Hoechst 33342 DNA intercalating agent can allow live monitoring of cell growth up to a certain point. Unfortunately, these strategies require treatments that affect cell physiology^1–4^. Furthermore, photo-bleaching of fluorescent tags render them useless for monitoring over several days.

To monitor cell growth in real time without interfering with the cells, we used Quantitative Phase Imaging (QPI), which is now well established^5–10^. QPI only requires short bursts of low intensity white light that are relatively harmless to living cells compared to fluorescence. QPI cameras produce phase images that are processed to extract biologically significant features. The optical phase differences measured after light propagates through biological samples are a direct reflection of the dry matter encountered along the optical path. Multiple reviews focus on this technique: among them Park et al., Kasprovicz et al., Zangle et al. and Popescu^11–14^. As others, we also showed previously that QPI accurately measures increases in the growing cell mass and gave methods to assess the accuracy of these measurements^5^. These and other features derived from QPI have now been associated with proximal machine learning methods to accurately classify cells in early S phase, late G2, and other phases of the cell cycle^15^.

In this work, we show that in addition to dry mass measurement, cell rounding associated to mitosis is also accurately detected by QPI. More importantly, we show that in addition to individual cells, whole cell populations can be monitored for their growth in a relatively straightforward manner by integrating the values of the features of interest from all the cells present in the acquired fields. This presents the advantage of averaging cell to cell disparities. In this way, even high density populations that are notoriously difficult to accurately segment can be monitored in real time for their growth. It is thus possible to perform non-invasive determinations of doubling time and real-time comparison of growth in multiple wells, even at high density.

## Results

### The principle of quantitative phase imaging

Data presented in this work rely on a wave front sensor camera (SID4Bio) based on Quadriwave Lateral Shearing Interferometry (QLSI)^16,17^. This camera was developed by PHASICS. It produces interferograms that are mathematically processed by the Biodata program, a R&D interface specifically developed for this study, first to analyze the interferograms and then to segment cells. Multiple features derived from Optical Path Difference measurement (OPD) are extracted for each identified cell. In addition to classical morphological features (surface, circularity, perimeter, …) relying on signal intensity usually derived from white light or fluorescence microscopy images, quantitative values are also obtained (phase value, dry mass, density…)^18^.

### Dry mass monitoring of a single live cell

**Figure 1:**
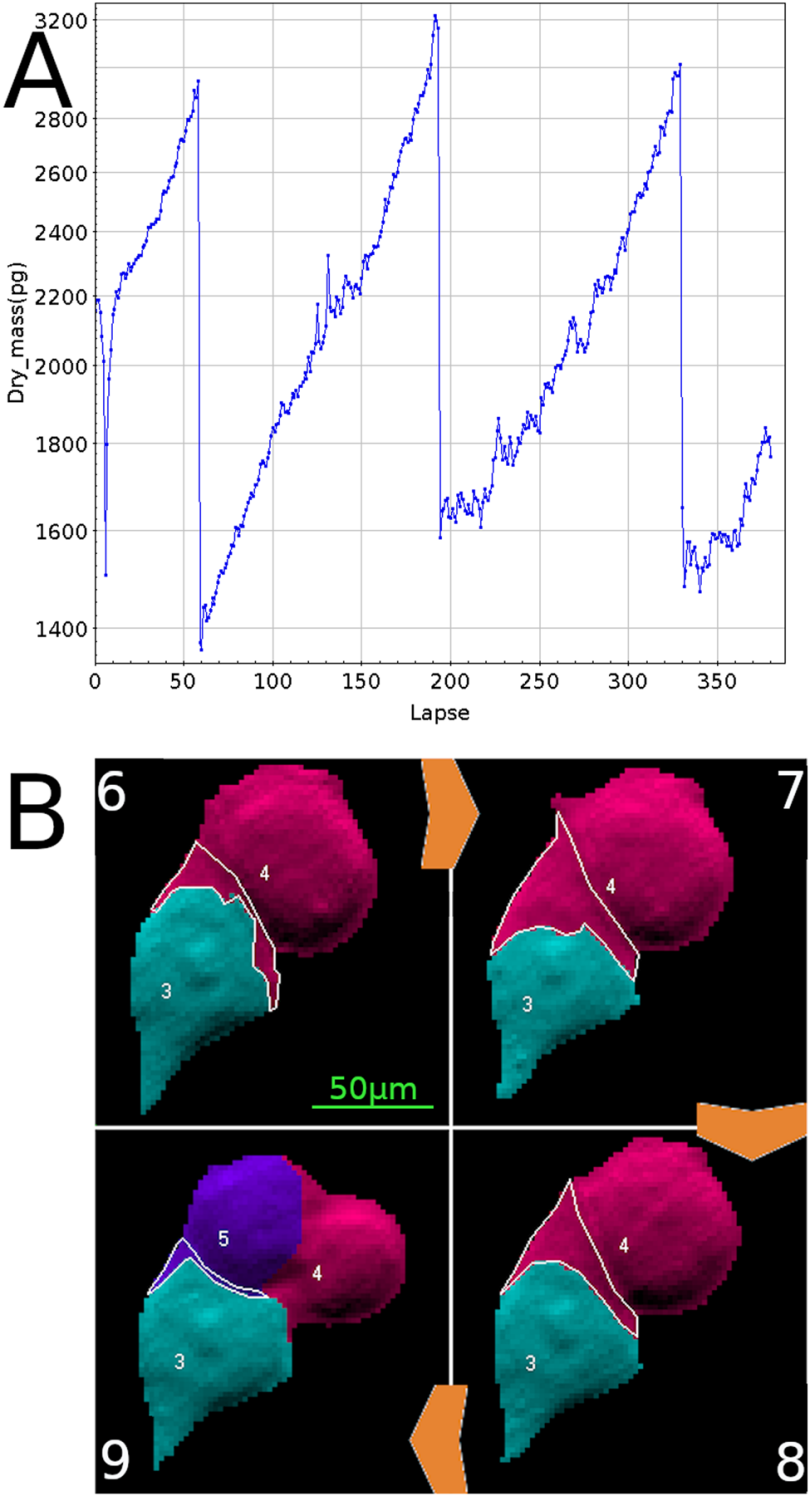
(**A**) 4-day time-lapse imaging of a single HEK cell. Dot-plot of the dry mass feature at every time point (15 min interval). The cell dry mass increases to a maximum value, and suddenly drops to a roughly half minimum value, corresponding to cell mitosis. It then slowly increases again until mitosis, and so on. (**B**) Detail of the cell being monitored (turquoise-colored cell) at lapses 6–7, when mitosis of the adjacent cell (burgundy-colored) results in a brief artifact in the segmentation of the monitored cell, resulting in the sudden but transient drop of dry mass seen at the beginning of the time-lapse. The "missing" dry mass is in the burgundy area circled by the thin white line. The picture field width is approximately 150 µm.

Cell monitoring with QPI has a long history. The work by Dunn and colleagues in 1989 already detected increases in single cell mass over several hours using interferometric microscopy^19^ and demonstrated that the spread area of chick heart fibroblasts is related to cell mass^20^. A change in the living cell dry mass could thus be automatically and accurately monitored over time, as illustrated on Fig. 1A. This plot shows the growth of an isolated cell placed in an incubation chamber (37 °C, 5% CO_2_, H_2_O saturated) under a microscope equipped with a QPI camera. All dot-plots presented in this work were produced with topcat, a very flexible, powerful and fast java application originally developed for handling data in the field of astronomy^21^. Topcat has been upgraded by its creator to also fit the needs of biologists. Figure 1A displays the dry mass of a single HEK cell, computed from the optical path differences recorded with the camera.

This cell was segmented and analyzed every 15 min (X-axis) for 4 days by time-lapse microscopy. A base-10 log scale was used for the Y-axis (dry mass), facilitating the visualization of the exponential gain of weight of this cell. The dry mass reaches a maximum and then suddenly drops to roughly a half-weight value (from ± 3000 pg down to ± 1500 pg). This sudden drop is due to mitosis, when the cell splits into two daughter cells. For easier presentation, the daughter cells are not shown here. One can see that mitosis occurs at lapse 59, 194 and 330, roughly every 135 lapses, i.e. every 33 h 45 min under this culture condition.

A sudden drop occurs at the beginning of acquisition, reaching a minimum at lapse 7. This is due to poor segmentation of the monitored cell that leaves part of the cell out, resulting in this artifactual drop. This type of artifact of segmentation is well known in the quantitative imaging field, and affects the accuracy of the measurement of the mass of single cells within dense and confluent cell populations, which we discussed in an earlier study^5^. This incorrect segmentation was due to a sub-optimal setting of the segmentation parameters that did not detect the physical hindrance caused by the ongoing mitosis of the flanking cell. This abruptly changed the geometry of the contact of the cell analyzed, resulting in this artifact. This is shown in Fig. 1B, where 4 BioData processed interferograms are shown. The four pictures are numbered with their lapse number. The top right hand picture ("7") shows the segmentation of cells #3 (bottom turquoise-colored cell, the one being monitored) and #4 (upper burgundy-colored cell) on lapse 7, when a sudden low dry mass was measured for cell #3 (Fig. 1A). The burgundy area delimited with a thin white line is actually part of cell #3 but wrongly attributed to its neighboring cell #4. This zone is smaller on lapses 6 and 8, and even smaller on lapse 9, when the burgundy daughter cell appeared as the violet-colored cell #5. These segmentation artifacts perfectly match the abnormal change in the dry mass observed on Panel A.

To summarize, data shown in Fig. 1 indicate that segmentation artifacts may severely impair the determination of single cell features. Nevertheless, regression curves on segmented cell time-lapse acquisition can provide an accurate growth rate of these individual cells as well as precise determination of mitosis. Using another quantitative feature (Phase Max, which is the maximum phase value inside the cell) we found that mitosis can also be very precisely detected in time-lapses of live cells by a sudden and sharp peak. This is shown in the Supplementary Information (Sup. Figures S1, S2).

However, since QPI image single cell mis-segmentation is less accurate when confluence increases, monitoring population growth becomes impossible after a few days in culture. We thus investigated an alternative method to accurately monitor population growth despite confluency.

### Integration of individual values of cell features to obtain information on population growth

**Figure 2:**
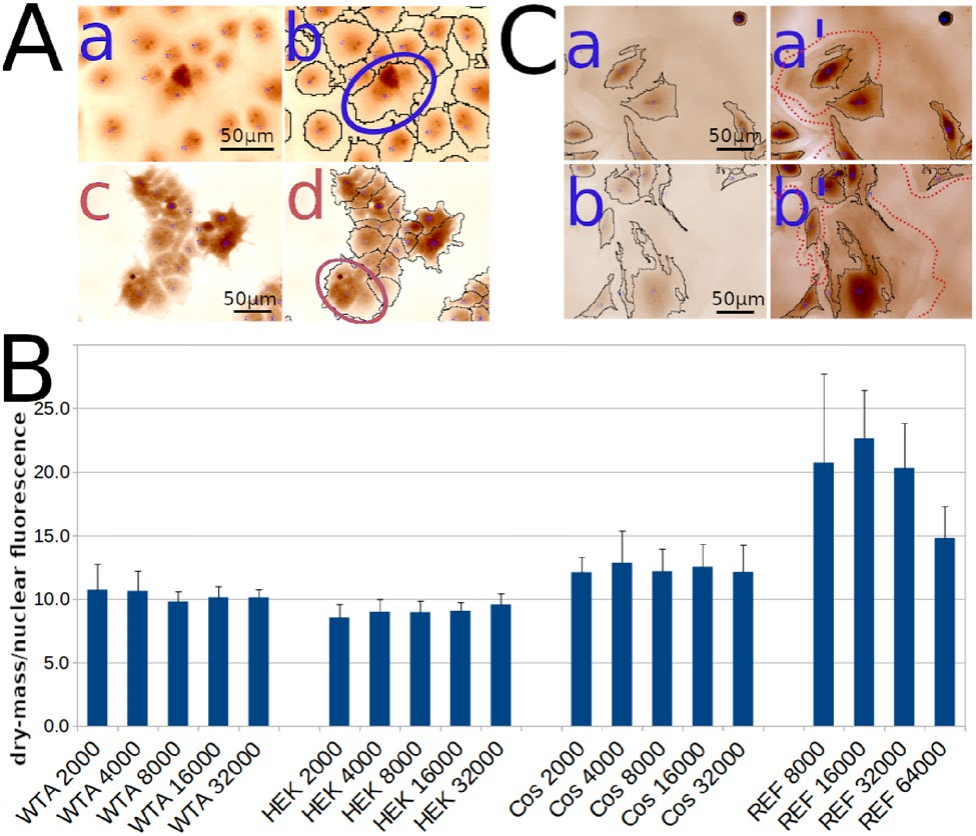
(**A**) Image a: Cos cells, segmented in b. The width of the picture is approximately 200 µm. Image c: HEK cells, segmented in d. The pictures are approximately 300 µm in width. For both Cos and HEK cells, some segmented areas encompass more than a single cell, as illustrated in the drawing of the 2 ellipses. However, for both cell types, all the cells present in the fields are within the segmented areas. (**B**) Bar graph representation of the ratio "integrated dry mass /integrated nuclear fluorescence intensity" for 4 different cell types at different plating concentrations. The error bars are the standard deviations calculated from the average of the Summed Dry mass/Total fluorescence ratios of all matching interferograms/fluorescent cell images analyzed in each of the 19 conditions presented in this experiment (4 cell types and 5 plating densities, except REF: 4 plating densities). There were from 12 to 24 independent interferograms/fluorescent images analyzed for each of the 19 conditions presented. Number of cells analyzed are shown in Supplementary Information, Table S1. (**C**) 2 examples of REF cell incomplete segmentation. a' and b' are contrasted version of a and b, respectively, where we can see that the cell periphery is barely detectable (circled with red dashed line). The picture field width is approximately 300 µm.

We developed a modification of the single cell monitoring approach presented above to monitor the growth of an entire cell population. We reasoned that since reliable segmentation of cells is necessary to obtain accurate measurements of single cells this could become a problem for global population monitoring. Indeed, it is not rare that growing cells tend to form tight foci in which segmentation becomes difficult, if not impossible. In addition, it is better to monitor a higher number of cells rather than a few found at 40 × magnification a few hours after seeding to measure the growth of a cell population. We thus selected a smaller magnification that in turn renders segmentation even more difficult due to frequent sub-segmentation (several cells seen by the segmentation program as a single cell). A way around sub-segmentation is to simply use a loose segmentation setting so that even if individual cell segmentation in a confluent situation may not be satisfactory/possible, all cells are nevertheless part of the segmented areas. The integration of all segmented areas present in a field gives a value similar to the sum of each individually segmented cells present in the same field. This is illustrated on Fig. 2A, for two different cell types acquired at 20 × magnification.

Images a and b are COS cells, that are quite easy to segment with Biodata even at 20 ×. Nevertheless, in the blue ellipse in the middle of the image, 3 cells have been segmented as one. Similarly, images c and d are of HEK cells that are notoriously difficult to segment when growing in foci. Our loose segmentation parameters result in tightly packed cells that are segmented as groups instead of individual cells (see red ellipse for example). However, all cells have been accurately integrated into the segmented areas (delimited by thin black lines), even if not as individual ones. Thus, the sum of the dry mass of these groups is a correct reflection of the sum of the dry mass of each individual cell.

To further demonstrate that even at the relatively low 20 × magnification the integrated dry mass over a field correlates well with the number of individual cells present in that field, we performed the following comparative experiment. Cells were incubated with Hoechst 33342. UV/blue wavelength illumination produces clear signals of nuclei on a black background of these Hoechst-labeled cells. These fluorescent nuclei were acquired with a CMOS camera and quantified using the Open Source CellProfiler program^22^. Since Hoechst is a DNA intercalating agent, the integrated fluorescent signal of a field is proportional to the number of cells present, assuming that each field from the same well has a similar ratio of 2 N/4 N. We reasoned that if the integrated dry mass of all segmented cell groups measured with QPI is indeed proportional to the number of individual cells present in the same field, then the ratio between the integrated dry mass and integrated Hoechst 33342 signal should be constant, independent of the number of cells present in the field under analysis. What we observed is presented on Fig. 2B, where each bar is, for the indicated condition, the average of between 12 and 24 different fields imaged at 20 × magnification with both a QPI and a CMOS camera. For each of the WTA, HEK and Cos cell lines, the ratio of the integrated dry mass divided by the integrated nuclear intensity are very homogeneous for 2000–32,000 inoculated cells per well of a 24 well plate. This is consistent with our hypothesis. The slightly different ratio from one cell type to another reflects the fact that nuclei from different cell lines do not produce the same Hoechst 33342 fluorescent signal, and/or do not have the same average QPI dry mass/cell. This is particularly true for REF cells, since we had to use a stronger excitation level to get a workable fluorescent signal. In addition, the REF “primary” cells grown here had already been culture for multiple passages and were not growing as well as the 3 lines used in parallel. The lowest plating that allowed REF cells to adhere and grow was 8000 cells/well of a 24 well plate, i.e. 4 times more than the 3 other cell lines, reflecting their reduced vitality. In addition, REF cells were very flat and widely spread, as expected from cells going towards senescence. Due to their very low mass/area in the cell periphery, their segmentation was not always complete, as seen on the two examples in Fig. 2C: a’ and b’ are force-contrasted versions of a and b, showing large and barely detectable cytoplasmic areas (surrounded with red dashed lines), that were similar to the background and consequently not well recognized. This explains the higher variability in the standard deviation and in the ratio of the integrated dry mass /integrated nuclear intensity for REF cells.

In conclusion, we showed that at least for immortalized cell lines, integration of dry mass values gives an accurate value of the number of cells imaged, strictly proportional to the number of nuclei detected by fluorescence. This integrated dry mass calculation does not require any treatment of the cells, leaving the cells completely unaffected during their monitoring, as opposed to Hoechst coloration, which is toxic to cells^3^. This cell dry mass determination can easily be transformed into a cell count by dividing the dry mass recorded by the average dry mass of individually segmented cells.

### Application to cell cultures exposed to a toxic drug

**Figure 3:**
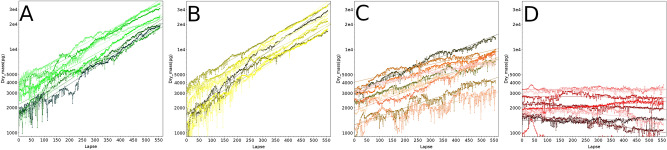
92.5 h-live monitoring of HEK populations starting on day 1 after addition of increasing staurosporine (STS) concentrations. Each line on these plots corresponds to the monitoring of one field encompassing multiple cells together with its linear fit (same color), and each STS concentration is monitored on 9 fields. (**A**) 4 nM STS, (**B**) 20 nM STS, (**C**) 100 nM STS, (**D**) 500 nM STS.

To validate our live cell population monitoring strategy, we first compared the growth of HEK cells cultured in the presence of increasing concentrations of staurosporine (STS), an alkaloid isolated from *Streptomyces staurosporeus*^23^. STS is highly toxic to cells due to its inhibitory activity on multiple kinases as well as on mitochondria, ultimately leading to apoptosis^24^. Figure 3A–D shows for each STS concentration tested the change in the integrated dry mass (Y axis, base-10 log scale) during 92.5 h (X axis, Lapse = 10 min) measured in 9 positions acquired at 20 × magnification, starting 24 h after adding STS to the culture medium. At t0, each field contained between 30 and 100 cells (thus different starting dry masses).

**Figure 4:**
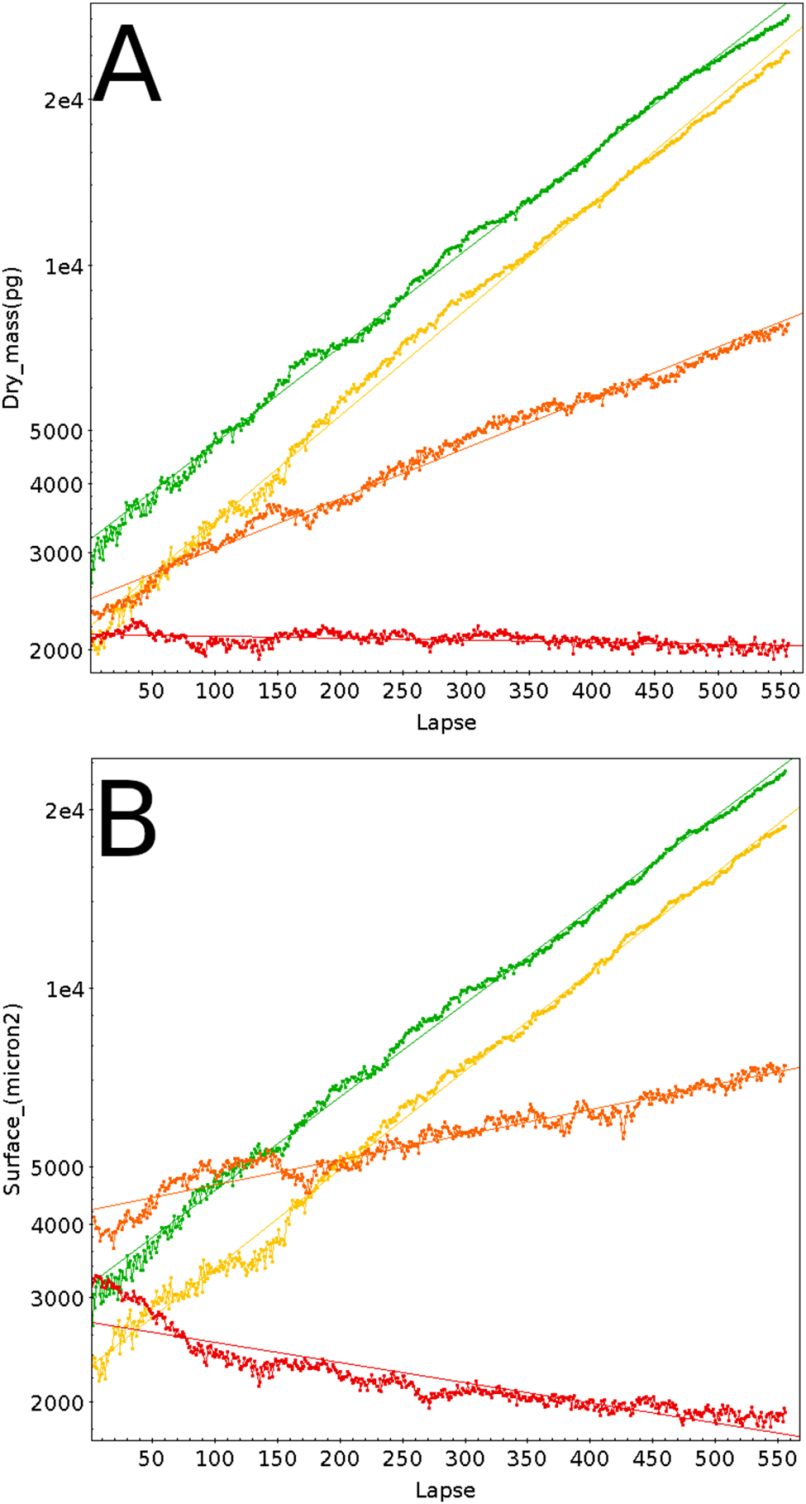
(**A**) Average of the 9 fields monitored for the 4 STS concentrations depicted in Fig. 3, with the same color code. Green: 4 nM STS, yellow: 20 nM STS, orange: 100 nM STS, red: 500 nM STS. (**B**)Same as (**A**), but using Surface features instead of Dry mass.

The position of each field monitored in a well is shown as a colored line, together with its corresponding linear fit (average correlation coefficients 0.99 for 4 and 20 nM STS, 0.95 for 100 nM and negative for 500 nM, due to cell loss). Colors on Panel A to D from green shades to red shades indicate the increasing STS concentrations. Figure 4 shows for each STS concentration (same color code) the averaged dry mass and cell surface measured for the 9 positions at each time point.

This demonstrates that 4 and 20 nM STS did not affect the growth of HEK cells in a dose-dependent manner (calculated doubling time close to 27 h for 4 and 20 nM STS) but affected cell growth at higher concentrations: 55-h doubling time for 100 nM, and “infinite” for 500 nM (cells die and did no longer divided).

It has been shown that the cell mass of a growing cell is directly related to its surface area^20^. To investigate whether this is also true for cells undergoing apoptosis, we performed the same experiment as the one shown in Fig. 4A, but using the Surface feature instead of Dry mass. This is presented in Fig. 4B. The similarity of the two images is striking. 100 and 500 nM STS resulted in an even more pronounced decrease in the cell surface than the decrease in mass as seen with the trend lines. The calculated doubling surface times are close to 31, 30, 118 h and “infinite” for 4, 20, 100, and 500 nM STS treatment, respectively. As visible for 500 nM STS during the ~ 100 first lapses, the cells undergoing apoptosis first shrunk without significant loss of integrity (nor significant loss of mass as seen on the left panel), to finally release apoptotic bodies and disintegrate. Our QPI data on populations of HEK cells cultured in the presence of STS is in good agreement with data originally obtained for HT29 treated with increasing STS concentrations^25^, where low STS concentrations (< 20 nM) did not induce apoptosis while high STS concentrations did.

To summarize, both the QPI Dry mass and Surface features provide reliable information to monitor cell populations grown under multiple conditions.

**Figure 5:**
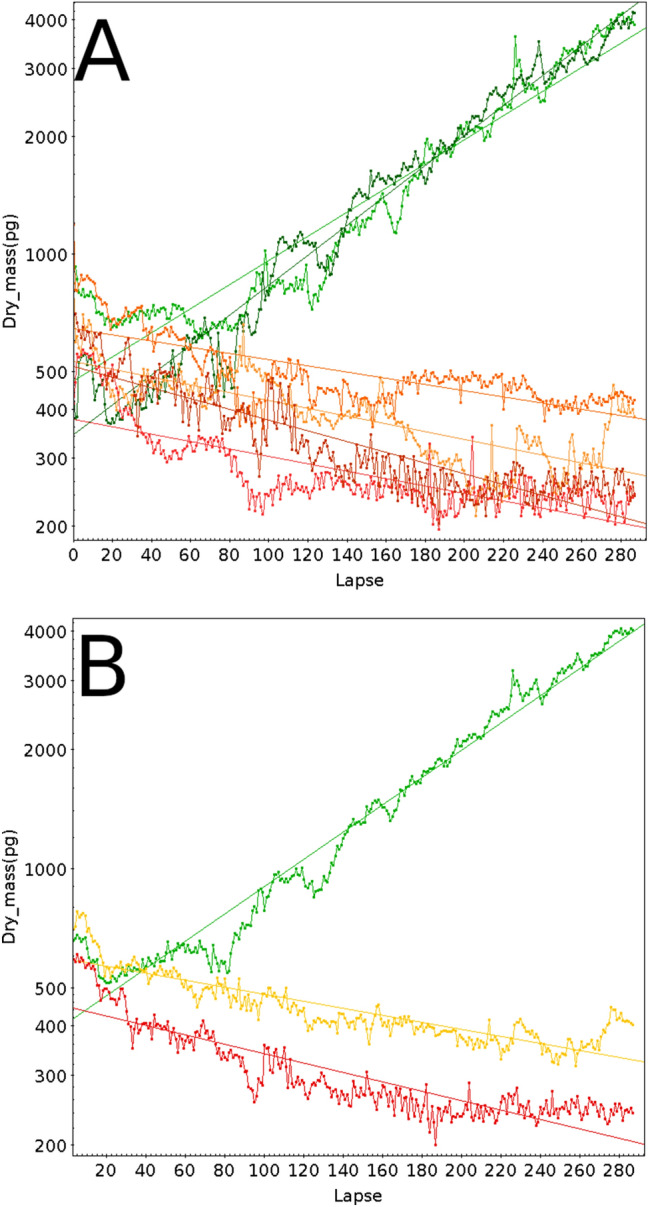
6-day live monitoring of REF populations in the presence of increasing staurosporine (STS) concentrations. Green: 0 nM STS; orange: 4 nM STS; red: 20 nM STS. (**A**) Each line on these plots corresponds to the monitoring of one field encompassing multiple cells together with its linear fit (same color), and each STS concentration was monitored on 2 fields with initial seeding of the 24-plate with 20,000 cells. (**B**) Average of the 2 fields of the 3 conditions monitored in (**A**), with the same color code. Green: 0 nM STS, orange: 4 nM STS; red: 20 nM STS.

As discussed above the very flat and adherent phenotype of REF cells make them difficult to accurately segment. As shown on Fig. 5A, Control REF cells (2 different positions, plotted in shades of green) are seen as jagged lines, due to the fluctuations in segmentation discussed earlier. Nevertheless the global trend in changes in the dry mass is clear and coherent. These cells were imaged right after seeding multi-well plates (105 cells/mm^2^), when cells start to adhere.

This is why a delay in growth was observed at the beginning of the time-lapse. We also performed this experiment in the presence of increasing STS concentrations. As little as 4 nM (orange colors) was enough to completely disrupt the cell growth and to lead to cell death, and all the more so with 20 nM STS (shades of red) at which the dry mass loss (cell death) occurred even sooner. As for the HEK cells above, we calculated the averaged dry mass measured at both positions for each time point, which is shown in Fig. 5B. The topcat calculated linear fit gave a 0.98 correlation for the REF control, and we calculated a 43-h 36 min-doubling time. The 4 and 20 nM STS linear fits were negative, indicating cell death, with as expected a faster loss of dry mass in the presence of 20 nM STS during the first 100 lapses.

To summarize, this strategy efficiently gave the doubling rate of cells in culture and allowed live monitoring of the effect of toxic compounds. We show here that late passage REF cells have a longer doubling time than HEK cells (44 versus 27 h), and that REF cells are much more responsive to low STS concentrations than HEK cells, since at 4 nM REF cells already die, whereas HEK cells keep on growing during at least 4 days up to 100 nM STS, but died at 500 nM STS, i.e., a > 100-fold resistance as compared to REF cells.

## Discussion

This work presents an efficient and robust method relying on light wave front analyses to monitor adherent live cell populations in a non-invasive manner. Any regular inverted microscope can be used, as long as it is equipped with a C mount to connect a camera. A QPI camera is the only specific piece of equipment needed. If lengthy time-lapse acquisitions are planned, the culture plate under the microscope must be in a controlled atmosphere. Many commercially available systems may be obtained for this purpose.

Other strategies have been proposed previously to quantitatively monitor living cells without affecting their growth, such as micro-electro-mechanical systems (MEMS) resonant mass sensors^7^. This method is able to follow single cells or small groups of cells in culture, and to obtain quantitative features from these cells. However, it requires very specifically designed sensors and uniform magnetic fields, which are not available in regular cell biology laboratories. While very interesting, it is unfortunately not an easy-to-implement method. Digital holography is another method based on interfering wave fronts from coherent light sources such as a laser^26^. While this approach also leads to quantitative feature measurements from a live cell culture, a specific integrated system combining a laser source and microscope is required. The authors of this approach mentioned that the 500 ms-laser illumination needed for each image has “only a minimal effect on the physiological functions of the cells”. While this may be the case, a few milliseconds exposure to a classical light bulb as the one of our standard inverted microscope will have even less effect, if any. We were actually unable to detect any difference between imaged and non-imaged cells in terms of cell growth and survival (data not shown). Furthermore, digital holography requires specific cell culture vessels for optimal results, while the approach described in our study works with regular thin bottom plates.

The most accurate determination of the weight of a living cell is undoubtedly obtained with the inertial picobalance method developed by Martinez-Martin et al.^27^. Their technique is so precise that the authors could show that the mass of a living cell oscillates within tens of seconds of 1–4% of its total mass, via a water exchange process. Using our QPI system, we measured the dry mass of growing HeLa cells every second for two minutes (data not shown). We could not detect any significant fluctuation, which is actually expected since the variations reported by the authors results from water exchange. Indeed, what we record with QPI was the cell dry mass. While the picobalance approach is remarkable, the exquisite sophistication of the micro-cantilever onto which a cell has to be transferred before its growth can be monitored makes the system hardly compatible with intensive everyday use to monitor the growth of multiple cells and cell-lines under multiple conditions, as can be done with our QPI approach.

The main restriction of our system is that cells must be adherent, since well focused cells are needed to obtain quantitative information. It is also preferable to use thin bottom plates to get optimal images. Finally, it is of course necessary to plate cells at a density compatible with exponential growth. Once these conditions are met, it is relatively straightforward to monitor single cells as well as cell populations. We confirm in this work that quantitative features provide accurate information regarding cell growth at the single cell level and show that growing cell populations at too high a density for accurate single cell segmentation can also be monitored with QPI. It is thus possible to detect subtle changes in cell growth without any labeling or cell manipulation. This system could be advantageously used for high-throughput screening (HTS) of banks of compounds when looking for modifications to growth, cell death or differentiation, since both quantitative and qualitative features obtained via QPI can distinguish these variations.

We currently process the QPI-produced interferograms after interferogram acquisition. Interferogram processing on the fly during time-lapse allows real-time monitoring of cell responses to the tested conditions. This saves time, specially when running HTS, since any effect could be detected as soon as the selected features in the treated cells start differing from the control cells. We are currently developing such an interface to display dot-plots of interest in real-time.

This method is also potentially valuable for people studying the impact of molecular mechanisms on cell physiology. Treatment of cells with toxic compounds, inhibitors, or transfection of cells with snRNA or expression vectors whose translation products are suspected to interfere with molecular processes would affect cell growth, phenotype or differentiation. QPI live imaging of treated cells will detect such modifications. This will be valuable for all types of screening tests.

## Materials and methods

### Imaging station

The imaging station used in this work is a Zeiss Axiovert 200 M microscope equipped with Definite Focus and a motorized Marzhauser Tango stage, set into an Okolab enclosure thermostated at 37 °C. A Pecon stage-top alimented with an Okolab gas mixer was used to maintain cell culture plates in water-saturated 5%CO_2_. A Zyla CMOS camera and a SID4Bio camera were installed on the C ports. The whole platform was driven by Open Source Micro-Manager software^28^.

### Quantitative phase imaging

In this work, we focus on the interest of using quantitative phase imaging to perform high content screening experiments.

#### Optical path difference

When light propagates through a sample, its interaction with matter creates local delays due to the change in its velocity by a factor named the refractive index. The accumulated delay relates to the phase of the light complex electromagnetic field:

$$A\left( {\vec{r}} \right) = \sqrt {I\left( {\vec{r}} \right)} e^{{j\varphi \left( {\vec{r}} \right)}}$$

where A is the field amplitude, I the field intensity and φ the field phase. In the projective approximation, where the field depth is much larger than the sample thickness, the field phase is related to the refractive index field n by

$$\varphi(\vec{r}) = \frac {2 \pi} \lambda \sqrt {n\left( {\vec{r}},z \right)}dz$$

where z is the propagation direction.

Phase is often identified to the Optical Path Difference W (OPD), independent of the wavelength defined as:

$$W = \frac{\lambda }{2\pi }\varphi = \smallint ndz$$

#### Phase imaging with a wave front sensor

The phase field is not easily revealed since standard cameras are only sensitive to the field intensity. Zernike first proposed to create contrast from phase information and DIC or Nomarski imaging is now widely implemented on microscopes. However, the phase value is not easily retrieved from these techniques. Digital holography^6,29^ was the first method to obtain quantitative phase images (QPI) in microscopy. A growing number of other techniques are currently implemented to measure phase (see^11^ for a review of the domain): Spatial Light Interference Microscopy (SLIM)^8^, Diffraction Phase Microscopy (DPM)^30^, Transport of Intensity Equation (TIE)^31^.

### QuadriWave lateral shearing interferometry (QLSI)

In this work, we proposed to use a wave front sensor to produce QPI images. A wave front sensor directly measures the phase and the intensity of the imaged field. Using a wave front sensor for QPI makes the implementation on a microscope straightforward since it replaces a standard video camera at any of the exit ports of a microscope and is compatible with any means of illumination. In addition, it is compatible with incoherent sources like halogen or LED sources, which drastically reduce noise. The wave front sensor used for these experiments is based on QuadriWave Lateral Shearing Interferometry (QLSI)^16,17^ and was developed by PHASICS (SID4 Bio). It improves the definition (i.e. the number of measurement points per camera pixel) of standard wave front sensing techniques like Shack-Hartmann.

The incident light field is sampled by diffraction grating, which generates four tilted replicas of the field (See illustration in Supplementary Information, Sup. Figure S3). They interfere on a camera chip placed a few millimeters downstream. The recorded camera image is a pattern made of a deformed grid where the fundamental period is the same as the diffraction grating period p.

It was shown^16^ that the grid deformation is proportional to the OPD spatial derivative $\frac{\delta W}{{\delta x}}$. The interference field is given by:

$$I{\text{int}}\left( x \right) = I\left( x \right)\left[ {1 + cos\left( {\frac{2\pi }{p}x + \frac{2\pi z}{p}\frac{\delta W}{{\delta x}}} \right)} \right]$$

where z is the distance between the grating and the camera chip. Here the equation is given in 1D for sack of simplicity whereas it can easily be extended to 2D.

The OPD gradient field is recovered from Fourier filtering around the fundamental frequency $\frac{1}{p}$ and finally numerically integrated. As seen in the equation above, the interferogram field is independent of the wavelength, which makes this technique achromatic, meaning it is compatible with white light and LED illumination.

### QPI features

To extract data from QPI images, phase images containing cells are first segmented (see next section). Then every cell is measured to determine the morphological and quantitative features (See illustration in Supplementary Information, Sup. Figure S4). Morphological features are those commonly used for white light or fluorescence images. They include cell surface, ellipticity, circularity, convexity and solidity.

Quantitative features are the analog to radiometric features and are related to the pixel values. It is important here to mention that pixel values are rather different from gray level values. The latter are only to be considered relatively, whereas quantitative phase imaging values are calibrated and relate to physical parameters, namely the local mass density (mass per unit of surface). Therefore values are consistent from one image to another. In this study, we used 6 QPI features: dry mass, average density, Max OPD, Mean OPD, OPD Median, OPD standard deviation. Other features describing mass inhomogeneities (texture features for instance) could also be included.

We here mentioned dry mass and density, which are not direct OPD features. However, the mass volume density ρ is proportional to the refractive index *n*:

$$n = n_{medium} + \alpha \rho$$

It was shown by Barer^32^ that α is almost constant for any intracellular components (lipids, proteins, DNA…).

$$\alpha \simeq 0.19pg/\mu m^{3}$$

Since the OPD is the integral of the refractive in one direction, it is proportional to the mass surface density. Therefore the average OPD inside the cell is rescaled by a factor α and is identified to the average mass surface density.

Similarly, if the OPD is integrated over the cell surface, we obtain a figure proportional to the cell dry mass. The cell dry mass is the mass of the cell excluding water, which is the surrounding medium.

$$m = \iiint \rho = \frac{1}{\alpha }\iint {OPD}$$

All steps from QPI image acquisition to growth rate data analysis presented here are also detailed in^5^, together with performance and limitations of our method to measure dry-mass from QPI images.

### Feature extraction method

For the purpose of this study, we developed automated software that processed camera images and generated data tables ready for analysis. The camera images (interferograms) were first converted into phase images using the algorithm mentioned above. The cells were then carefully segmented in two steps. First we detected the background areas from the cell areas. The criteria for discrimination is based on variance: background parts have much lower phase variance than the cell parts. In other words, cells are considered to be in areas where the signal-to-noise is the highest.

In the "cell" part, we segmented individual cells using the assumption that each individual cell looks like a hill. Cell boundaries were identified as the lowest part between the hills. A watershed algorithm was used to detect them and separate the different cells.

The background part of the image contains some information used to accurately evaluate the cell dry mass. As seen before, the dry mass was determined by integrating (summing) the phase values inside each segmented cell. For this calculation to be accurate, the phase values should not be biased. However, interferometric methods only give access to phase values with an unknown offset. Therefore phase images are naturally biased. Moreover, due to variation in the optical quality of coverslips in each dish, phase variations occur within each field of view. These variations usually have low spatial frequencies. This adds some uncertainty to the estimation of the dry mass.

To unbias and compensate for phase spatial variations, we considered that the background should be an area where phase values vanish. We made a polynomial fit of the phase values in the detected background area, extended the polynomial values to the whole image and subtracted the obtained image from the phase image. This new image was then used to estimate the dry mass. We finally assessed our assumption by calculating the variance of the residual signal in the background area. If it is larger than usual values, this indicates that there are errors in the camera image or that the image quality is poor. Such fields and the data were removed from subsequent data analyses.

Finally, the morphological and QPI features were extracted from the segmented cells, using bias-corrected values.

### Cell culture

Cells were plated in 1 ml of culture medium in Ibidi thin-bottom 24-well plates at 10,000 cells per well, otherwise indicated. This results in an average of 53 cells/mm^2^. At that density, cells are separate, allowing for exponential growth. Culture media were 0.45 µm filtered to remove floating debris that could produce artifacts during acquisition. All cells were grown in DMEM 10% FCS without any antibiotics. STS was solubilized in DMSO, and intermediate dilutions were prepared extemporaneously in PBS. 10 µl was added per well to reach the indicated final concentration. The cells used were Human Embryonic Kidney HEK-293 cells from ATCC (CRL-1573), HEK-293 cells expressing SLC5A5, the sodium-iodide symporter (NIS): WTA cells, Monkey epithelial cells expressing SV40 Large T antigen: COS cells, and late passage (> 20) primary rat embryo fibroblasts (REF).

### Data analysis

Following Biodata processing of interferograms, data tables consisting of 20 columns (Acquisition\_Name, Cell\_ID, Surface(micron2), Phase\_PV(nm), Phase\_StdDev(nm), Phase\_avg(nm), Phase\_median(nm), Surface\_density(pg\/micron2), Dry\_mass(pg), Optical\_volume(micron3), Phase\_max(nm), Phase\_min(nm), Circularity, Perimeter(micron), Ellipse\_axis\_ratio, Convexity, Solidity, Surface\_Centroid\_X, Surface\_Centroid\_Y, Mass\_Centroid\_X, Mass\_Centroid\_Y) and as many lines as cells analyzed were produced. These tables were processed with the Open Source topcat software, which is both versatile and powerful^20^. Topcat was used for the exploration of the different features presented above by dot-plot visualization in the search of the most discriminant ones (dry mass and phase max in this work).

Hoechst labeled nuclei were quantified using the Open Source software CellProfiler^21^. Nuclei Integrated Intensity (per image) feature was selected for the calculation of the "integrated dry mass /integrated nuclear intensity" ratio reported in Fig. 2B.

All experiments presented in this work were performed at least three times.

## Supplementary Information

Supplementary Information 1.

## Abbreviations

QPI: Quantitative phase imaging
DNA: Deoxyribonucleic acid
S phase: Synthesis phase
late G2 phase: Late gap2 phase
SID4Bio: Name of the wave front camera used in this work
QLSI: Quadriwave lateral shearing interferometry
OPD: Optical path difference measurement
ATCC: American type culture collection
HEK: Human embryonic kidney
WTA: HEK-293 cells expressing the sodium-iodide symporter
REF: Rat embryo fibroblast
COS: Monkey epithelial cells expressing SV40 Large T antigen
HeLa: Human cervix epithelial adenocarcinoma
DMEM: Dulbecco's modified eagle medium
FCS: Fetal calf serum
BioData: Program developed to process interferograms
Phase Max: Maximum phase value inside the cell
CMOS: Complementary metal oxide semiconductor
STS: Staurosporine
MEMS: Micro-electro-mechanical systems
HTS: High-throughput screening
SLIM: Spatial light interference microscopy
DPM: Diffraction phase microscopy
TIE: Transport of intensity equation
DMSO: Dimethyl sulfoxide

## References

[1] Ansari AM (2016). Cellular GFP toxicity and immunogenicity: potential confounders in in vivo cell tracking experiments. Stem Cell Rev.

[2] Ganini D (2017). Fluorescent proteins such as eGFP lead to catalytic oxidative stress in cells. Redox Biol..

[3] Purschke M, Rubio N, Held KD, Redmond RW (2010). Phototoxicity of Hoechst 33342 in time-lapse fluorescence microscopy. Photochem. Photobiol. Sci..

[4] Bucevičius J, Lukinavičius G, Gerasimaitė R (2018). The use of Hoechst dyes for DNA staining and beyond. Chemosensors.

[5] Aknoun S (2015). Living cell dry mass measurement using quantitative phase imaging with quadriwave lateral shearing interferometry: An accuracy and sensitivity discussion. J. Biomed. Opt..

[6] Kemper B, von Bally G (2008). Digital holographic microscopy for live cell applications and technical inspection. Appl. Opt..

[7] Park K (2010). Measurement of adherent cell mass and growth. Proc. Natl. Acad. Sci..

[8] Mir M (2011). Optical measurement of cycle-dependent cell growth. Proc. Natl. Acad. Sci. USA.

[9] Kim T, Popescu G (2011). Laplace field microscopy for label-free imaging of dynamic biological structures. Opt. Lett..

[10] Girshovitz P, Shaked NT (2012). Generalized cell morphological parameters based on interferometric phase microscopy and their application to cell life cycle characterization. Biomed. Opt. Express.

[11] Park Y, Depeursinge C, Popescu G (2018). Quantitative phase imaging in biomedicine. Nat. Photon.

[12] Kasprowicz R, Suman R, O’Toole P (2017). Characterising live cell behaviour: Traditional label-free and quantitative phase imaging approaches. Int. J. Biochem. Cell Biol..

[13] Zangle TA, Teitell MA (2014). Live-cell mass profiling: an emerging approach in quantitative biophysics. Nat. Methods.

[14] Popescu, G. *Quantitative phase imaging of cells and tissues*. (McGraw-Hill, 2011).

[15] Pognonec, Philippe *et al.* Non invasive live cell cycle monitoring using quantitative phase imaging and proximal machine learning methods. in *2019 IEEE 32nd International Symposium on Computer-Based Medical Systems (CBMS)* 483–488 (IEEE, 2019). doi:10.1109/CBMS.2019.00099.

[16] Bon P, Maucort G, Wattellier B, Monneret S (2009). Quadriwave lateral shearing interferometry for quantitative phase microscopy of living cells. Opt. Express.

[17] Primot J, Guérineau N (2000). Extended Hartmann test based on the pseudoguiding property of a Hartmann mask completed by a phase chessboard. Appl. Opt..

[18] Aknoun S, Bon P, Savatier J, Wattellier B, Monneret S (2015). Quantitative retardance imaging of biological samples using quadriwave lateral shearing interferometry. Opt. Express.

[19] Brown AF, Dunn GA (1989). Microinterferometry of the movement of dry matter in fibroblasts. J. Cell. Sci..

[20] Dunn GA, Zicha D (1995). Dynamics of fibroblast spreading. J. Cell. Sci..

[21] Taylor M (2017). TOPCAT: desktop exploration of tabular data for astronomy and beyond. Informatics.

[22] Carpenter AE (2006). Cell Profiler: Image analysis software for identifying and quantifying cell phenotypes. Genome Biol..

[23] Omura S (1977). A new alkaloid AM-2282 of Streptomyces origin taxonomy, fermentation, isolation and preliminary characterization. J. Antibiot..

[24] Park B (2013). Staurosporine analogues from microbial and synthetic sources and their biological activities. CMC.

[25] Qiao L (1996). Staurosporine inhibits the proliferation, alters the cell cycle distribution and induces apoptosis in HT-29 human colon adenocarcinoma cells. Cancer Lett..

[26] Mölder A (2008). Non-invasive, label-free cell counting and quantitative analysis of adherent cells using digital holography. J. Microsc..

[27] Martínez-Martín D (2017). Inertial picobalance reveals fast mass fluctuations in mammalian cells. Nature.

[28] Edelstein AD (2014). Advanced methods of microscope control using μManager software. J. Biol. Methods.

[29] Cuche E, Bevilacqua F, Depeursinge C (1999). Digital holography for quantitative phase-contrast imaging. Opt. Lett..

[30] Popescu G, Ikeda T, Dasari RR, Feld MS (2006). Diffraction phase microscopy for quantifying cell structure and dynamics. Opt. Lett..

[31] Phillips KG, Jacques SL, McCarty OJT (2012). Measurement of single cell refractive index, dry mass, volume, and density using a transillumination microscope. Phys. Rev. Lett..

[32] Barer R (1952). Interference microscopy and mass determination. Nature.

