# Supplementary material for: Quantitative phase microscopy for non-invasive live cell population monitoring
Source: Sci Rep. 2021 Feb 24;11:4409. doi: 10.1038/s41598-021-83537-x (PMC7904828; doi:10.1038/s41598-021-83537-x)
Supplement: Supplementary file 1 — Supplementary Information 1. [file 41598_2021_83537_MOESM1_ESM.pdf]

Supplemental Data

Quantitative phase microscopy for non-invasive live cell population monitoring

Sherazade Aknoun [a1]; Manuel Yonnet [a1]; Zied Djabari [b]; Fanny Graslin [b]; Mark Taylor [c]; Thierry Pourcher [b]; Benoit Wattellier [a]; Philippe Pognonec [b]*

Sup. Figure 1 :

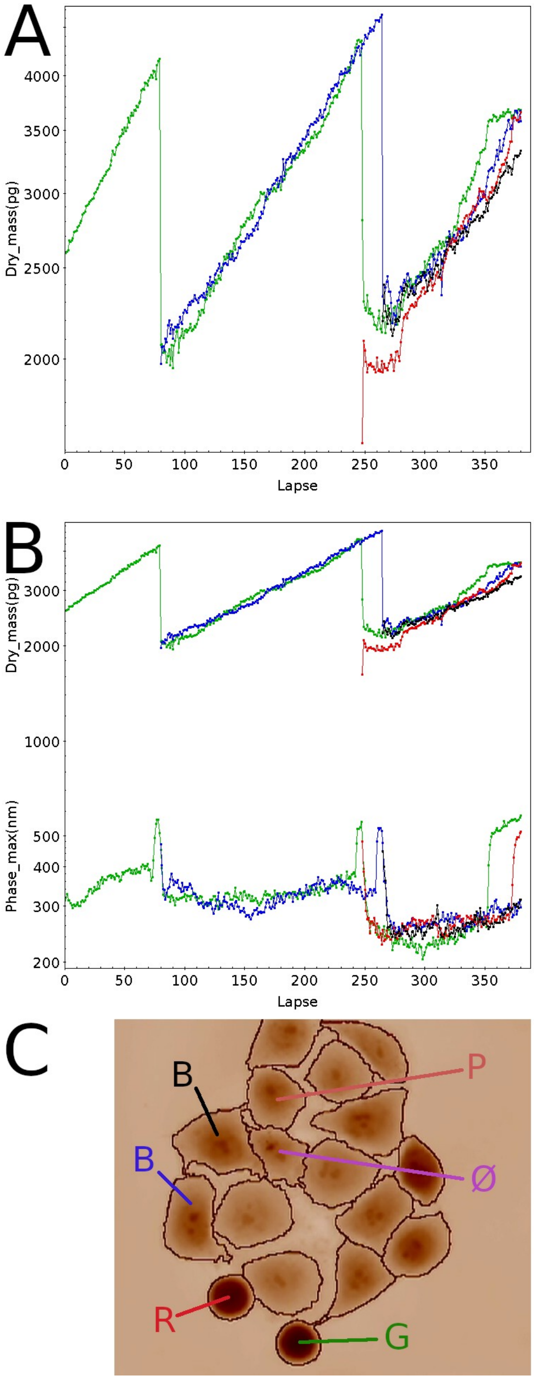

Panel A, 4-day time-lapse QPI monitoring of a single HEK cell and its daughter cells. Panel A, the "green" cell dry mass increases to a maximum value, then drops to an approximately half minimum value, corresponding to the cell mitosis. At that point, the daughter cell appears as a blue line, and dry masses increase until the next mitoses, where two additional daughter cells appear (black and red lines).

Panel B, the upper part is dry mass as in Panel A, while the lower part displays another feature called Phase maximum, which corresponds to the highest value of phase in the segmented cell. Transient and well defined peaks are seen for this feature at each cell division. It is interesting to see that cell cycle synchronicity is lost right away after division.

Panel C, the position of the 4 cells monitored at the end of the time-lapse, using the same color code (G: green, B (blue): blue, R: red, B(black): black. P and Ø point to cells described in more detail in Sup. Fig. 2. Each BioData segmented cell is delimited by a thin black line. The picture field width is 285µm.

Sup. Figure 2 :

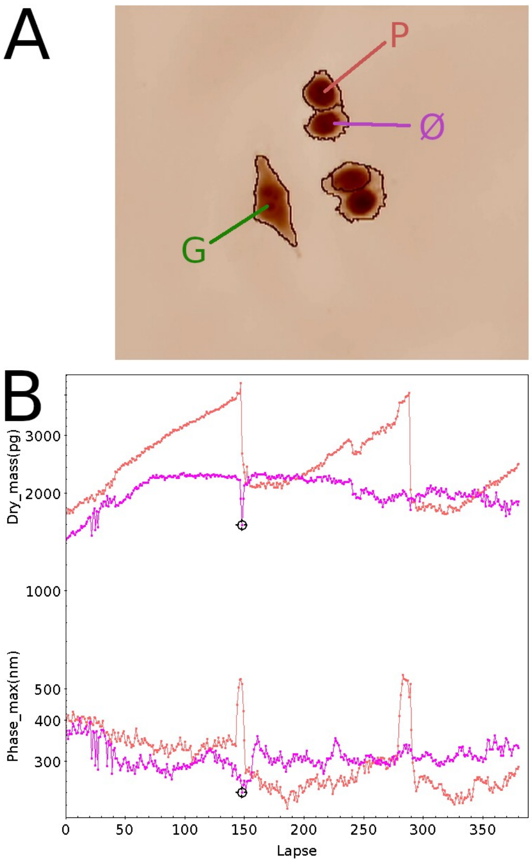

Panel A, 4-day time-lapse imaging of two HEK daughter cells. The cells labeled P and Ø are two daughter cells at the beginning of the time-lapse. The picture field is 285µm.

Panel B, the upper part is dry mass of these two daughter cells presented in Panel A. The lower part is another feature from these same two cells: The phase maximum, which corresponds to the highest value of phase in the segmented cells. Transient and well defined peaks are seen at each "red cell" division, but none for the "purple cell", which stopped growing, as seen on the Sup. Video.

Sup. Figure 3 :

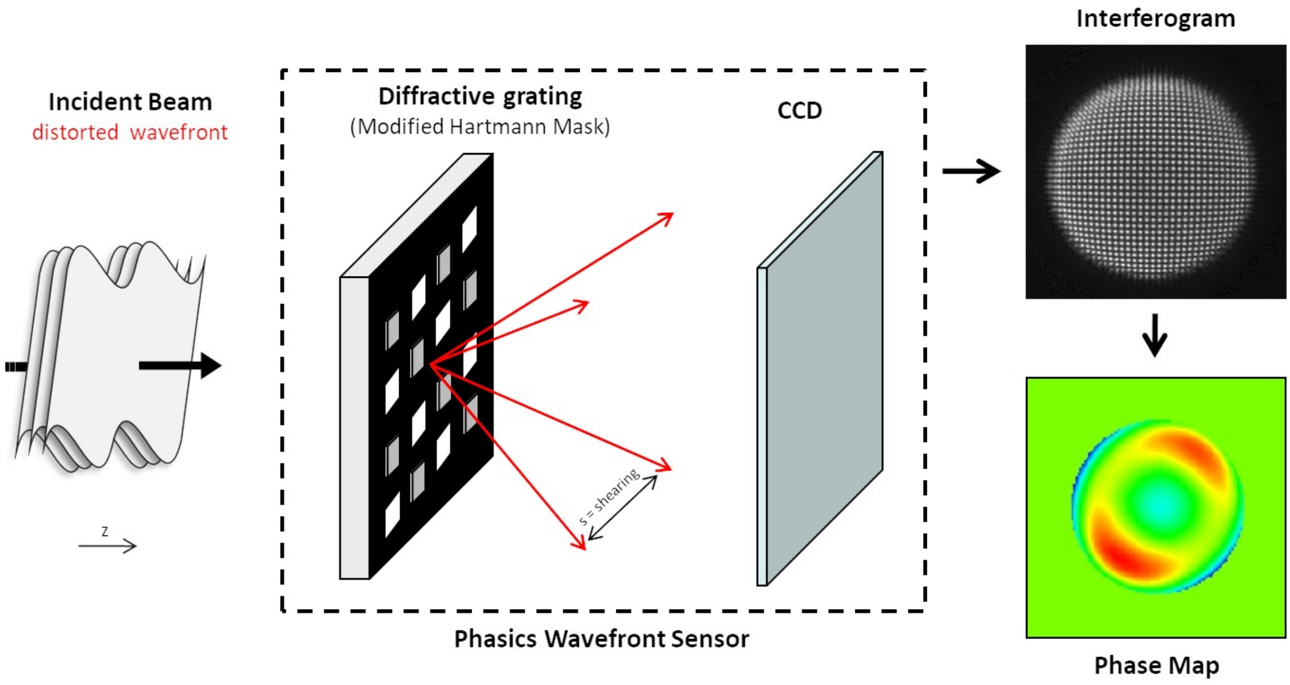

QLSI principle. The light impinging from the left diffracts through a diffraction grate and then interferes on the camera detector (CCD). The diffraction grating is based on the Modified Hartmann

Mask principle, where a grid of holes is phase modulated by π every other hole. Interference fringes that make the interferogram are deformed by phase gradients. The Phase Map is derived from the Interferogram.

Sup. Figure 4 :

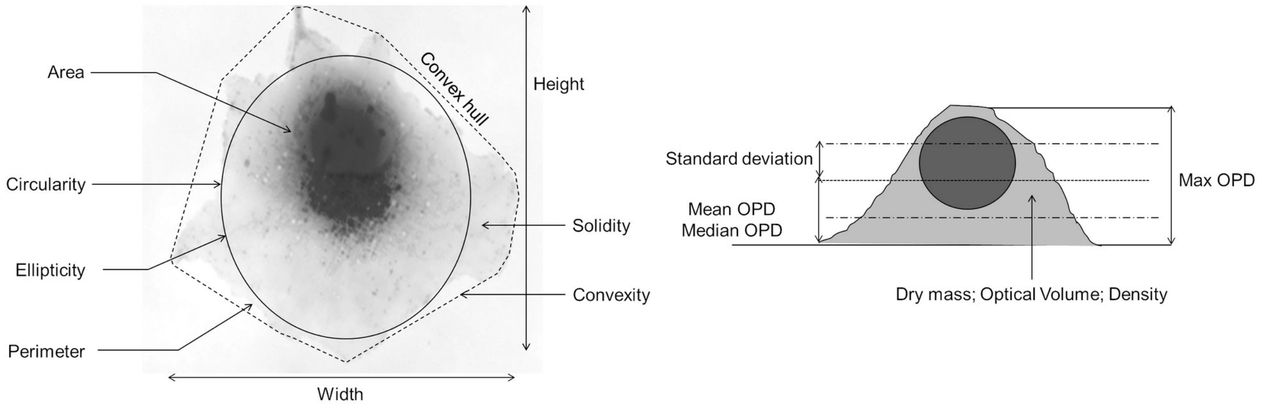

Features extracted from QPI images of segmented cells. Left: Morphological features as defined in CellProfiler software21 . Right: QPI features based on QPI image pixel values.

Sup.Table 1:

Plated cells	Cell number analyzed
WTA 2000	194
WTA 4000	659
WTA 8000	1375
WTA 16000	2321
WTA 32000	2783
HEK 2000	233
HEK 4000	528
HEK 8000	1102
HEK 16000	2147
HEK 32000	5018
Cos 2000	170
Cos 4000	601
Cos 8000	1334
Cos 16000	1380
Cos 32000	1876
REF 8000	55
REF 16000	111
REF 32000	476
REF 64000	2587

This table recapitulates the number of cells analyzed in Figure 2.

Sup. Movie :

BioData recording of the growing population from Sup. Fig.1 and Sup. Fig.2, in which the “green cell” (lowest cell in Sup. Fig.2 Panel A) is blocked in metaphase, visible from 1 min 10 sec till the end of this time-lapse video. The positions of the 4 cells monitored in Sup. Fig.1 Panel A and B at the end of this time-lapse are shown in Sup. Figure 1, Panel C.
